# Adverse Childhood Experiences and Education Outcomes among Adolescents: Linking Survey and Administrative Data

**DOI:** 10.3390/ijerph191811564

**Published:** 2022-09-14

**Authors:** Ashley Stewart-Tufescu, Shannon Struck, Tamara Taillieu, Samantha Salmon, Janique Fortier, Marni Brownell, Mariette Chartier, Alexa R. Yakubovich, Tracie O. Afifi

**Affiliations:** 1 Faculty of Social Work and Children’s Hospital Research Institute of Manitoba, University of Manitoba, Winnipeg, MB R3T 2N2, Canada; 2 Department of Community Health Sciences, University of Manitoba, Winnipeg, MB R3E 0W3, Canada; 3 Department of Community Health Sciences, Manitoba Centre for Health Policy, and Children’s Hospital Research Institute of Manitoba, University of Manitoba, Winnipeg, MB R3T 2N2, Canada; 4 Department of Community Health & Epidemiology, Dalhousie University, Halifax, NS B3H 1V7, Canada; 5 Departments of Community Health Sciences and Psychiatry, Children’s Hospital Research Institute of Manitoba, University of Manitoba, Winnipeg, MB R3T 2N2, Canada

**Keywords:** adverse childhood experiences (ACEs), child maltreatment, education, adolescents, administrative data, survey data, the Well-Being and Experiences Study

## Abstract

It is well established that adverse childhood experiences (ACEs) are associated with detrimental health outcomes in adulthood. Less is known about the relationships between ACEs and education outcomes and among adolescents. The aim of this study was to examine the associations between ACEs and adolescents’ self-reported education outcomes and provincial education assessments among adolescents in Manitoba, Canada. Data were gathered from 1002 adolescents who participated in the Well-Being and Experiences (WE) Study. A subsample of the adolescents (84%) consented to having their WE survey data linked to administrative education databases. Binary and multinomial logistic regression models were computed to examine associations between ACE history and self-reported education outcomes and provincial education assessments, adjusting for sociodemographic variables. Adolescents with an ACE history had significantly increased likelihood of having ever been suspended from school (adjusted odds ratio (aOR) = 3.33, 95% CI 1.60–6.92), of lower grades (adjusted relative risk ratio (aRRR) = 3.21, 95% CI 1.42–7.29), and of chronic school absenteeism (aRRR = 2.45, 95% CI 1.28–4.68) compared with adolescents without an ACE history after adjusting for sociodemographic variables. Findings from this study illuminate the important relationship between childhood adversity and poor education outcomes assessed directly by adolescents. Increasing awareness of the public health risk associated with ACEs and education outcomes may inform education policy and school-based interventions.

## 1. Introduction

It has been over two decades since the seminal Adverse Childhood Experience Study first identified the relationship between childhood adversity and the leading causes of morbidity and mortality among a sample of adults from the United States [1]. Since then, numerous high-quality studies, including meta-analyses and systematic reviews, have detailed the robust associations between adverse childhood experiences (ACEs)—traditionally defined as ten distinct forms of child maltreatment and household challenges—and numerous detrimental health outcomes in adulthood [2,3,4]. These include internalizing and externalizing disorders [5,6]; physical health conditions [4,7]; suicidal behaviours [8,9]; and poor well-being and functioning [3,10]. Despite this substantial body of literature detailing the relationships between ACEs and detrimental health outcomes in adulthood, little is known about the impacts of ACEs among adolescents and young adults and on other domains of health and well-being such as education.

This dearth of information is confirmed in a recent bibliometric analysis of two decades of ACE publications (1998 to 2018) that concluded that the ACEs literature represents a thriving field of study, one that is rapidly expanding beyond the realm of medical domains, mental and physical health outcomes, adult samples from primarily Western contexts, and traditional ACEs [11]. This includes a shift towards multidisciplinary domains; broader social outcomes related to well-being; child, adolescent, and intergenerational samples; populations from diverse geographical contexts; and expanded and contemporary experiences of ACEs. Following this transition, one emerging area of research aims to understand the impacts of ACEs on educational engagement and academic achievement. The few previous studies that have examined this relationship have found a history of ACEs to be associated with poor education outcomes related to academic success [12,13], school engagement [14], school drop-out [15], and school attendance [16]. School-aged children residing in the United States with a history of ACEs (defined as two or more ACEs) had increased odds of poor education outcomes, including lower school engagement, more specialized individual education plans, more school absenteeism, and more grade retention (i.e., repeating a grade) compared with school-aged children without a history of ACEs [14]. In a related study utilizing administrative education data from Minnesota, children with a history of child protection involvement including documented child maltreatment and exposure to intimate partner violence (IPV) were found to have low scores in reading and mathematics [17]. While a notable strength of this study was the use of administrative data to examine the impact of child maltreatment and IPV on academic performance among school-aged children, it remains unknown whether similar trends in education assessments would be found among a more general population of adolescents without a previously identified history of child protection involvement but with a history of ACEs and from diverse geographical contexts.

In recent years, leading ACE scholars have increasingly called for the critical assessment of the definition, conceptualization, and measurement of ACEs [18,19,20,21] and for the inclusion of children and youth populations in ACE research [11]. However, with few exceptions, the limited studies that have examined associations between ACEs and education outcomes have relied primarily on adults’ and school personnel’s reporting of children’s history of adversity and education outcomes [12,13,16,22], and have utilized variations of the original 10-item ACEs questionnaires [12,23]. While the term ACEs is widely conceptualized as five forms of child maltreatment (i.e., physical, sexual, and emotional abuse, physical and emotional neglect) and five forms of household challenges (i.e., mother treated violently, household member substance abuse, household member mental illness, parental separation or divorce, and household member incarceration), recently there have been calls to expand the list of ACEs to examine other adverse experiences that are theoretically and empirically similar to the traditional 10 ACE items and are associated with poor health outcomes [24]. Previous research that aimed to improve the rigour associated with the measurement of ACEs compared the impacts of the original ACEs items with additional adverse experiences on health outcomes and concluded that the additional ACEs examined (e.g., peer victimization) explained more variance associated with poor health outcomes than the original ACEs [18,19]. Similarly, a confirmatory factor analysis of the original ACE study data from the United States found that spanking loaded onto the child maltreatment factor and explained additional variance related to mental health impairment outcomes including substance misuse and suicidality [25]. Building on this knowledge, ACE research has moved beyond a reliance on the original 10 ACE items and has expanded to include childhood experiences of spanking/physical punishment, bullying/peer victimization, involvement with the child welfare system, parental gambling, perceived neighbourhood safety, and poverty [24,25,26,27,28]. Including this expanded list of theoretically and empirically derived ACEs provides the opportunity to assess a broader range of collective adverse experiences on education outcomes in a Canadian context, which has not been examined in the literature previously.

The majority of the research on the impact of ACEs is retrospective in nature and relies on adults recalling their childhood experiences of adversity [11]. Little is known about children’s and adolescents’ own self-reported experiences of childhood adversity. It was only in 2017 that child and youth self-reported ACEs first appeared in the ACE literature [11]. This gap in our understanding may be partially explained by research ethics considerations and mandatory child abuse reporting requirements when directly studying children’s and adolescents’ experiences of adversity during childhood [29]. Relying on school personnel’s reporting of children’s adversity histories may reduce burden and minimize risk for the child and their family [12], but it remains questionable how accurately school personnel are able to assess children’s lifetime experiences of child maltreatment and household challenges without in-depth knowledge of the children’s families and home life circumstances. Examining adolescents’ own self-reported adversity history and their educational experiences may help to elucidate the relationship between ACEs and education outcomes during childhood rather than solely relying on adult reporting of adolescents’ ACEs and education outcomes. In sum, directly investigating adolescents’ perspectives of their experiences of adversity during childhood, and their self-perceived education experiences and outcomes, is another important avenue of research.

Taken together, there is still much to learn about the relationships between experiences of childhood adversity and education outcomes including academic achievement and school engagement among adolescents from geographical locations outside of the United States. While a few notable studies have begun to bring attention to the detrimental impacts of childhood adversity on education outcomes over the life course [12,13,14,15,16,22,23], we are not aware of any previous studies that have utilized an expanded list of adolescent self-reported ACEs and examined the associations between an ACE history and a variety of education outcomes garnered from multiple data sources. Thus, the overall purpose of the present study was to address these notable gaps in the literature by examining the association between adolescent self-reported ACEs and various education outcomes in a Canadian context using linked administrative and survey data. The specific aims of this research were to describe the histories of ACE exposure and education outcomes reported by adolescents and to determine if adolescents with a self-reported history of ACE exposure had an increased likelihood of poor education outcomes compared with those without an exposure history.

## 2. Materials and Methods

### 2.1. Data and Sample

Data for this study are from the Well-being and Experiences (WE) Study, an intergenerational and longitudinal cohort study of parent–adolescent dyads from Winnipeg, Manitoba, a mid-sized Canadian city, and surrounding communities. A detailed description of the WE Study recruitment methods and study design has been published previously [24,27,28]; however, in brief, the sampling design used random-digit dialling (21%) and convenience sampling (79%), including referrals and community advertising. No differences were found for several variables between groups based on recruitment methods (e.g., household income, ACEs). The Forward Sortation Area from postal codes was used to ensure that the sample was a close approximation of Winnipeg, Manitoba [24,27,28]. During Wave 1 of the study, we recruited 1002 adolescents aged 14–17 years and one of their parents to participate. Parents and adolescents separately provided consent to participate and independently completed a self-administered computer questionnaire at a research centre between July 2017 and October 2018. For the purpose of this study, only the Wave 1 (baseline) data collected from the adolescents were examined. A subsample of the adolescent responses from Wave 1 was linked to administrative data held in the Manitoba Population Research Data Repository (the Repository) at the Manitoba Centre for Health Policy (MCHP). For this step, 84% of the adolescent respondents provided their Personal Health Identification Numbers (PHINs) and consented to have their WE survey data linked to administrative databases at MCHP. The WE survey data were then linked to the administrative data using scrambled, de-identified PHINs following established, validated methods routinely used at MCHP [30,31]. A sensitivity analysis found no differences in adolescent ACE history, sex, or household income between adolescents who provided consent for data linkage and those who did not with the exception of age (refer to Supplementary Table S1).

### 2.2. Measures

#### 2.2.1. Adverse Childhood Experiences (ACEs)

ACE history was assessed with a series of 13 constructs/items that represent experiences of adversity during childhood including maltreatment, household challenges, and peer victimization (see Appendix A—Table A1 for a detailed description of the constructs and related items). The 13 ACEs were selected based on empirical evidence from a previously published confirmatory factor analysis that found the items to load onto the same conventional two factors (the child maltreatment factor and the household challenges factor) as the original 10 ACE items [24]. For the purpose of this research, an adolescent was considered to have an ACE history if they indicated having experienced one or more of the following: (i) emotional abuse, (ii) emotional neglect, (iii) exposure to IPV, (iv) household substance use, (v) household mental health issues, (vi) parental separation or divorce, (vii) parental problems with police, (viii) spanking (10 years of age and younger), (ix) peer victimization, (x) household gambling problems, (xi) foster care placement or child protective organization (CPO) contact, (xii) poverty, and/or (xiii) neighbourhood safety. An ACE history variable was created by combining these 13 ACE constructs/items.

#### 2.2.2. Education Outcomes

Education outcomes were measured using both WE Study survey questions and administrative provincial educational assessments held within the Repository. The WE survey included several questions regarding education outcomes, including adolescent self-reported grades, grade repetition, school absenteeism, school suspensions, and educational aspirations.

Provincial educational assessments completed by teachers in grade 3, grade 7, and grade 8 in the areas of numeracy, literacy, mathematics, reading, writing, and school engagement were obtained for the subsample of adolescents who provided their PHINs for data linkage purposes. Unlike standardized testing, these comprehensive classroom-based formative (assessment *for* and *as* learning) and summative (assessment *of* learning competencies) assessments were conducted to assess individual students’ strengths and challenges in order to plan individual student learning, guide instructional plans, and provide feedback about the level of attainment on a standard set of curriculum-specific competencies to the student and their caregivers, the school, and the division [32]. The teacher conducted these assessments over a period of weeks during daily teaching instruction at standardized times in the school year and incorporated multiple sources of evidence to increase the reliability and validity of the assessment of the student’s learning [32]. These assessments were submitted electronically by the teacher to the provincial department of education and included in the student’s cumulative education file. These province-wide assessments have been conducted in all provincially funded schools throughout the province of Manitoba consistently since 2009/2010. Data from these provincial grade-level educational assessments have been used in previous studies [33,34,35]. Details of the self-reported education questions and the specific competencies examined in the provincial assessments by grade level are presented in Appendix A—Table A1.

#### 2.2.3. Sociodemographic Covariates

We included the following as covariates in the analyses: sex, household income, and age. Due to limited variation in age per grade, age was not included as a covariate in the adjusted models for the grade-specific provincial assessments.

### 2.3. Statistical Analysis

Descriptive statistics and binary and multinomial logistic regression models were run to investigate the associations between lifetime ACE history among adolescents and education outcomes. Models were first unadjusted and then adjusted for sociodemographic variables. Statistical analysis of the survey data was performed using STATA 16, (StataCorp2019, College Station, TX, USA), and the administrative data held within the Repository at MCHP were analysed using SAS 9.4 (SAS Institute Inc., Cary, NC, USA). Following STATA 16 documentation (2019), binary logistic regression models are computed and reported as odds ratios, whereas multinomial logistic regression models are computed and reported as relative risk ratios.

This study received ethics approval from the Health Research Ethics Board at the University of Manitoba, and data access permission was granted by the Government of Manitoba’s Health Information and Privacy Committee and by Manitoba Education.

## 3. Results

Characteristics of adolescents self-reporting one or more ACEs compared with those with no ACE history are presented in Table 1. Female adolescents and adolescents living in a lower-income household more often reported an ACE history than male adolescents and adolescents from more affluent households. A self-reported ACE history was associated with lower self-reported grades, higher prevalence of school absenteeism and school suspensions, and lower (and less certain) longer-term educational aspirations.

The odds or relative risk ratios of self-reported education outcomes for adolescents with at least one ACE compared with those with no ACEs for the sample of adolescents (linked and not linked surveys) are provided in Table 2. After adjusting for age, sex, and household income, adolescents with at least one ACE had significantly increased odds of having ever been suspended from school (Model 2, adjusted odds ratio [aOR] = 3.33, 95% confidence interval [CI] = 1.60–6.92) compared with adolescents with no ACE history. Adolescents with an ACE history also had a significantly increased relative risk of reporting having grades of C or lower (vs. mostly As; adjusted relative risk ratio [aRRR] = 3.21, 95% CI = 1.42, 7.29) and being absent from school 4 or more days (vs. 0 days; aRRR = 2.45, 95% CI = 1.28, 4.68) in a typical month compared with adolescents without an ACE history.

The results for the sub-analysis of adolescents with self-reported survey data linked to administrative data (n = 840) are provided in Table 3. Significant differences by ACE history were found for competencies in grade 7 numeracy and student engagement; however, these effects were attenuated and no longer significant after adjusting for sex and household income. No other statistically significant differences were noted on grade-specific provincial assessments based on adolescents’ self-reported ACE history after adjusting for sex and family household income level. A sensitivity analysis was performed to examine dose–response trends with these data based on coding the ACEs as 0 ACEs, 1 ACE, 2 ACEs, 3 ACEs, and 4+ ACEs. Results did not differ substantially, and dose–response trends were found for many of the education outcomes assessed, although it should be noted that some models in the supplementary analyses are likely underpowered, as noted by some medium to large effect sizes and wide confidence intervals. Type II errors are possible, and findings should be interpreted accordingly. These results are presented in the Supplementary Materials section as supporting information.

## 4. Discussion

While there is a robust body of literature that has found ACEs to be associated with poor health outcomes in adulthood, there remains a great deal to learn about the impacts of ACEs on other important aspects of an individual’s well-being such as education. Moreover, there is a gap in our understanding of children’s and adolescents’ perceived experiences of ACEs, and among populations residing in diverse geographical locations other than the United States, as well as of the influence of other experiences of childhood adversity on important outcomes in addition to the typical ACEs studied. Thus, the purpose of this research was to address these notable limitations in the literature by uniquely examining the relationships between ACEs and education outcomes among adolescents residing in a Canadian context using administrative and self-report survey data. The findings from this study add to the literature in several important ways. In this study, having an ACE history was associated with lower grades, more school absenteeism, and more school suspensions after adjusting for sex and household income. These findings may be used to inform the development and implementation of school-based interventions for children and adolescents with a history of ACEs, education policy, and ACE prevention initiatives in the hopes of bolstering positive education outcomes while increasing quality of life, well-being, and upward social mobility for children with a history of adversity [36].

From a public health perspective, education is an important social determinant of health that is associated with life expectancy, morbidity, and health behaviours. Consistent with prior research from the United States [12,15,16,17,23,37], our findings extend and further refine the developing body of literature demonstrating that adolescents with an ACE history (i.e., one or more ACEs) are more likely to experience chronic school absenteeism and lower academic achievement. In addition, this work offers two novel contributions to the literature, finding that an ACE history is associated with school suspensions (even after adjusting for sociodemographic characteristics) and lower educational aspirations in a Canadian setting. These findings also extend our understanding of the influence of childhood adversity on education outcomes including school attendance and future aspirations to obtain higher education. Increasing awareness of the risks associated with childhood adversity in the North American education context and through education policy is critical to preventing school dropouts, which may have important implications for broader poverty reduction strategies and mental health initiatives over the life course and across generations [36].

This study was also able to examine associations between childhood adversity and education outcomes using an expanded and contemporary list of 13 ACEs including physical punishment, child welfare involvement, peer victimization, parental gambling, poverty, and perceived neighbourhood safety. Prior research has found this expanded list of ACEs to be associated with poor health outcomes including adolescent substance use [27] and now poor education outcomes. While mandatory Canadian child abuse reporting and ethics requirements prevented us from asking adolescent respondents directly about some types of maltreatment such as physical abuse and neglect, this expanded list of ACEs may indeed serve as proxy indicators for some of these experiences localized to this geographical setting.

Linking administrative education databases to adolescent self-reported survey data provided a unique opportunity to examine the relationship between ACEs and provincial education assessments in elementary and middle school in this Canadian context. Unlike previous research that used administrative data from the United States to examine the impact of child maltreatment on academic achievement among younger children [17], in our study, adolescents’ history of ACEs was only found to be associated with poorer academic achievement in grade 7 mathematics and school engagement competencies prior to adjusting for sociodemographic variables. One explanation for this finding may be related to how these provincial educational indicators were created and analysed. It may be the case that additional differences in provincial education assessments by ACE history would be identified if the grade-level, subject-specific competencies were examined independently (e.g., ‘met expectations’, ‘approaching expectations’ and ‘not yet meeting expectations’), rather than dichotomously (e.g., ‘meeting and approaching expectations’ versus ‘not meeting expectations’). Another explanation may be related to the nature of the educational assessments. In Manitoba, Canada, provincial education assessments are primarily intended for planning for student learning and are not intended to be used for standardized testing, although they do indicate a level of student achievement as assessed by the educator. Moreover, had we directly assessed physical abuse and neglect, rather than using proxy indicators, we may have found similar results to a previous study [17]. Interestingly, while not pronounced in the provincial assessment analyses, adolescents with an ACE history compared with those without an ACE history were more likely to self-report lower grades than higher grades, suggesting that childhood adversity is indeed associated with self-reported lower academic achievement. It may be the case that the detrimental impacts of ACEs take longer to manifest and may not be captured in the provincial educational assessments conducted in elementary and middle school. Future research may provide opportunities for a more nuanced approach to assessing the impacts of ACEs on education outcomes during high school and postsecondary levels in this context. This is of particular importance given that our study found significant differences by ACE history related to grade 7 competencies (i.e., numeracy and student engagement), although these results were lessened after adjusting for sex and household income and reported wide confidence intervals. These results should be viewed as preliminary and warrant future research with larger samples to better understand the impacts of ACEs on meeting provincial education expectations in a wide variety of grade- and education-related domains (i.e., literacy, numeracy).

A major strength of this study was adolescents’ direct participation in this research rather than relying on parents’/caregivers’ or school personnel’s reporting of adolescents’ childhood adversity histories and education outcomes, as has been the case previously [12,13]. Based on this approach, some of the most illuminating findings from this study are the associations between self-reported history of ACEs and education outcomes including future educational aspirations, self-reported grades, and school absenteeism and suspensions that parents/caregivers and other informants may not be privy to or able to accurately report on. Furthermore, adolescents have a right to participate in matters which concern them, including research [38]. More is necessary in this regard to reasonably balance rights to protection from harm with rights to directly participate in research about their lives.

The findings of this study should be considered in the context of its limitations. While adolescents were asked to report on ACEs before the age of 16 years, it is unknown when the adverse experiences occurred. Thus, we cannot know if the ACEs preceded the education outcomes, and causality should not be inferred from these findings; rather, is an important avenue for future research. Adolescents were not asked to report on specific experiences of abuse and neglect (i.e., physical or sexual abuse, physical neglect) but instead to report on proxy indicators due to mandatory Canadian child abuse reporting laws and ethics requirements. This may have underestimated the relationships between ACE history and education outcomes. Similarly, adolescents’ self-reported ACE history may have been underestimated if they were unwilling and/or unable to report their experiences of childhood adversity. Another potential limitation was the recruitment strategy and the context from which the study population was drawn. While this was a non-randomly generated sample of adolescents that is closely representative of the general population of Winnipeg and surrounding communities [24], caution should be used when generalizing the findings to different geographical contexts and education systems and among diverse populations. Lastly, while a notable strength of this study was the analysis of adolescent self-reported survey data linked to administrative education databases, not all of the adolescents in this study consented to having their data linked. Additional limitations that may be addressed in future research with larger samples may include examining ACEs individually and examining ACE dose–response relationships, including frequency and duration of ACEs associated with provincial education assessments, conducting longitudinal studies with more specific and narrowly defined experiences of adversity during childhood, and exploring resiliency factors that may reduce the vulnerability of children with an ACE history. Another avenue for future study is to conduct additional research on the conceptualization and operationalization of experiences of childhood adversity. This may contribute to increased specificity of how these experiences are defined and measured in contemporary society, where separation and divorce, financial constraints, and mental health issues such as parental depression and anxiety are increasingly common and recognized.

Based on these findings, one important avenue for future research is to investigate potential protective factors that may attenuate the risk of poor education outcomes for adolescents with an ACE history residing in similar geographical locations. This may include assessing protective factors in the educational context such as the quality of the student–teacher relationship, students’ sense of belonging and connection to the school community and environment, and other important protective factors such as the quality of the parent–child relationship and children’s secure attachment to their caregivers. Previous research has found these factors to reduce risk among adolescents with an ACE history in other important domains (i.e., substance use), and they may also be important in fostering better education outcomes [28]. Moreover, the identification of protective factors may assist in the development of school-based interventions for children and adolescents with a history of childhood adversity that may be at risk for academic challenges in similar geographical contexts and social locations. Further research would also benefit from directly engaging with adolescents to learn more about *how* early adversities have an impact on important domains of well-being with lifelong consequences. Elevating the inclusion of adolescents’ voices in research may also act as a conduit to involving adolescents in the development and critique of education and public health policy that has a direct impact on their well-being.

## 5. Conclusions

Education is an important social determinant of health and is related to income, employment, and career opportunities with significant implications over the life course. Knowledge of the relationship between childhood adversity and poorer/lower education outcomes is of relevance to public health leaders, child-serving professionals, education administration personnel, and other policy makers seeking to improve education outcomes for children who may be at risk academically. These results may inform the development and implementation of interventions aimed at reducing poorer education outcomes among adolescents with a history of childhood adversity.

## Figures and Tables

**Table 1:** Characteristics of adolescents with and without at least one ACE.

	≥1 ACE (n = 764) %	No ACE (n = 123) %	*X^2^* (df)/*t* (df)
Sex			
Male	45.47	56.91	5.57 (1) *
Female	54.53	43.09	
Household income			
$49,999 or less	24.55	0.88	51.72 (3) ***
$50,000–$99,999	37.72	29.20	
$100,00–$149,999	20.85	38.05	
$150,000 or more	16.87	31.86	
Age			
Years, mean (sd)	15.32 (1.09)	15.21 (1.05)	1.06 (884)
Self-reported grades			
A	55.57	73.77	20.46 (2) ***
B	22.15	20.49	
C or lower	22.28	5.74	
Grade repetition			
No	93.33	97.54	3.25 (1)
Yes	6.67	2.46	
Absenteeism (monthly)			
0 days	25.44	37.40	14.37 (2) ***
1–3 days	48.15	50.41	
4 or more days	26.40	12.20	
In-school or out-of-school suspension			
Never	78.86	92.68	13.03 (1) ***
1 or more	21.14	7.32	
Educational aspirations			
High school or less	19.16	7.32	19.48 (2) ***
Any post-secondary	71.52	90.24	
I don’t know	9.32	2.44	

ACE = adverse childhood experience; * *p* ≤ 0.05; *** *p* ≤ 0.001.

**Table 2:** Odds or relative risk of poor education outcomes for adolescents with at least one ACE compared to those with no ACEs (n = 887).

Education Outcome	Model 1—Unadjusted OR (95% CI)	Model 2—Adjusted OR (95% CI)
Grade repetition		
No	reference	reference
Yes	2.83 (0.87, 9.23)	1.72 (0.50, 5.93)
In-school or out-of-school suspension		
Never	reference	reference
1 or more	3.40 *** (1.69, 6.84)	3.33 *** (1.61, 6.92)
	RRR ^a^ (95% CI)	adjusted RRR (95% CI)
Self-reported grades		
A	reference	reference
B	1.43 (0.89, 2.32)	1.11 (0.67, 1.84)
C or lower	5.16 *** (2.34, 11.36)	3.21 ** (1.42, 7.29)
Absenteeism (monthly)		
0	reference	reference
1–3 days	1.40 (0.92, 2.14)	1.44 (0.91, 2.28)
4 or more days	3.18 *** (1.72, 5.90)	2.45 ** (1.28, 4.68)
Educational aspirations		
High school or less	3.30 *** (1.64, 6.68)	1.93 (0.92, 4.03)
Any postsecondary	reference	reference
I don’t know	4.82 ** (1.49, 15.58)	3.34 * (1.00, 11.13)

ACE = adverse childhood experience; OR = odds ratio; CI = confidence interval; RRR = relative risk ratio; models adjusted for sex, age, and household income, ^a^ Stata computes relative risk ratio and not odds ratios for multinomial logistic regression; * *p ≤* 0.05; ** *p ≤* 0.01; *** *p ≤* 0.001.

**Table 3:** Odds of education outcomes on provincial assessments for adolescents with at least one ACE compared with adolescents with no ACEs.

Provincial Grade Level Assessments	≥1 ACE %	No ACEs %	Model 1—Unadjusted OR (95% CI)	Model 2—Adjusted OR (95% CI)
Grade 3 Numeracy (Meeting/approaching)	83.08	16.92	0.67 (0.33, 1.36)	0.83 (0.39, 1.73)
Grade 3 Reading (Meeting/approaching)	83.47	16.53	0.75 (0.33, 1.72)	0.99 (0.41, 2.36)
Grade 7 Mathematics (Meeting/approaching)	84.01	15.99	0.39 (0.18, 0.83) *	0.50 (0.23, 1.09)
Grade 7 Student Engagement (Established/developing)	84.16	15.84	0.54 (0.29, 0.99) *	0.58 (0.30, 1.09)
Grade 8 Reading/Writing (Meeting/approaching)	85.27	14.73	0.72 (0.30, 1.74)	1.04 (0.40, 2.69)

ACE = adverse childhood experience; OR = odds ratio; CI = confidence interval; adjusted for sex and household income; * *p* ≤ 0.05.

## Data Availability

The Well-being and Experiences data included in this article are not publicly available due to REB protocols for this study.

## Supplementary Materials

The following supporting information can be downloaded at: https://www.mdpi.com/article/10.3390/ijerph191811564/s1, Table S1: Group differences between adolescents linked to administrative data at MCHP and those who did not consent to linkage; Table S2: Odds and relative risk ratios of the independent variable and covariates in the adjusted regression models (Model 2 in Table 2) for the self-reported education outcomes for adolescents with at least one ACE compared with those with no ACEs; Table S3: Odds ratios of the independent variable and covariates included in the adjusted regression analyses (Model 2 in Table 3) for the provincial educational assessment outcomes for adolescents with at least one ACE compared with those with no ACEs; Table S4: Odds or relative risk of education outcomes for adolescents with 1, 2, 3 or 4+ ACEs compared with adolescents with no ACEs.; Table S5: Odds of education outcomes for provincial assessments for adolescents with 1, 2, 3, or 4+ ACEs compared with adolescents with no ACEs.

## Appendix A

**Table A1:** Description of Study Measures and Operationalization of Variables.

	ACEs Variable (Independent Variable)	
ACE Construct	WE Survey Questions and Response Options	Operationalization
Emotional abuse	How many times in the past 12 months has a parent or other adult living in your home said hurtful or mean things to you? Response options: ‘everyday’, ‘several times a week’, ‘once a week’, ‘a couple times a month’, ‘once a month’, ‘several times a year’, ‘a few times a year’, ‘once a year’, ‘never’	Emotional abuse was indicated if the respondent noted that it occurred ‘once a month’ or more often.
Emotional neglect	Five items from the Childhood Trauma Questionnaire (CTQ) [39]	Emotional neglect was indicated if the summed score of the 5 items was 15 or greater, representing moderate to severe neglect.
Exposure to verbal intimate partner violence	How often in the past 12 months have you ever seen or heard adults say hurtful or mean things to another adult in the home?	EIPV was indicated if the respondent noted it occurred ‘once a month’ or more often.
Household substance abuse	Has a parent or other adult in your home ever had problems with alcohol or spend a lot of time drinking or being hung over? Has a parent or other adult living in your home ever had problems with drugs? Response options: Yes/No	Household substance use was indicated if the respondent noted ‘yes’ to one or both items.
Household mental health issues	Has a parent or other adult living in your home ever had. mental health issues like depression or anxiety? Response options: Yes/No	Household mental health issues was indicated if the respondent noted ‘yes’ to this item.
Parental separation or divorce	Have your biological parents ever been separated or divorced? Response options: Yes/No	Parental separation or divorce was indicated if the respondent noted ‘yes’ to this item.
Problems with the police	Has a parent or other adult living in your home ever had problems with the police? Response options: Yes/No	Parental problems with the police was indicated if the respondent noted ‘yes’ to this item.
Spanking	In a typical year, when you were 10 years or younger, about how often do you remember an adult spanking you with their hand on your bottom (bum)? Response options included: ‘never’, ‘less than once a year’, ‘once a year’, ‘two to three times a year’, ‘four to five times a year’, ‘six to 11 times a year’, ‘about once a month’, ‘about two times a month’, ‘about once a week’, ‘several times a week’, ‘daily’	Spanking was endorsed if the respondent indicated it occurred ‘two to three times a year’ or more often before the age of 10.
Parental gambling	Has a parent or other adult living in your home ever had problems with gambling? Response options: Yes/No	Parental gambling was endorsed if the respondent indicated ‘yes to this item.
Foster care or CPO contact	Have you ever been placed in a foster home or group home by Child and Family Services? Have you ever seen or talked to anyone from a child protective organization (like social services, child welfare, children’s aid, or the Ministry) due to difficulties at home? Response options: Yes/No	Foster care or CPO contact was endorsed if the respondent indicated ‘yes’ to either or both items.
Poverty	How often does your family run out of money or find it hard to pay for rent or mortgage on your house? How often does your family run out of money or find it hard to pay for basic necessities like food or clothing? Response options: ’never’, ‘rarely’, ‘sometimes’, always’, often’, ‘very often’	Poverty was endorsed if the respondent indicated ‘sometimes’ or more often to one or both of the items.
Neighbourhood safety	How much do you agree with the following statement: I feel safe in my community. Response options: ‘strongly disagree’, ‘disagree’, ‘neither agree nor disagree’, ‘agree’, ‘strongly agree’	Unsafe neighbourhood was endorsed if the respondent ‘disagreed’ or ‘strongly disagreed’ with the statement.
Peer-victimization	How many times in the past 12 months has a friend, peer, kid at school, or other young person (not an adult or a sibling) done any of the following to you: Made fun of you, called you names or insulted you in person or behind your back, but excluding texting, email, social media, or online posting or communications. Spread rumours about you in person or behind your back, but excluding texting, email, social media, or online posting or communications. Pushed you, shoved you, tripped you, or spit on you. Said something bad about your race, culture, or religion in person or behind your back, but excluding texting, email, social media, or online posting or communications Said something bad about your sexual orientation or gender identity in person or behind your back, but excluding texting, email, social media, or online posting or communications. Said something bad about your body shape, size, or appearance in person or behind your back, but excluding texting, email, social media, or online posting or communications. Bullied, picked on you, or said mean things about you, or threatened you through texting or the Internet (e.g., posted something on Facebook or other social media, or sent texts or emails). Response options: ‘never’, ‘1 or 2 times a year’, ‘3–6 times a year’, ‘7–11 times a year’, ‘once a month’, ‘a couple times a month’, ‘once a week’, ‘a couple times a week’, ‘every day’	Peer victimization was indicated if any of the seven items were experienced by the respondent monthly or more often in the past 12-month period assessed.
Education outcomes (dependent variables)		
Adolescent self-reported	WE Survey Education Questions	
Self-reported grades	On your last report card, how would you describe your grades in school? If you are no longer in school, please describe your last report card before leaving school.” Response options included: a) mostly 80% to 100% (mostly A’s), b) Mostly 70% to 79% (mostly B’s), c) Mostly 60% to 69% (mostly C’s), d) Mostly 50% to 59% (mostly D’s), and e) Mostly less than 50%.	Responses were grouped into 3 categories- mostly As, mostly Bs, and mostly Cs or lower
Grade repetition	Respondents were asked if had “ever repeated a grade (including kindergarten)?” Response options: Yes/No	Grade repetition was endorsed if the respondent indicated ‘yes’ to this item.
School absenteeism	“In a typical month during the school year, about how many days are you absent from school for any reason?” Response options: ‘0 days’, ‘1 to 3 days’, ‘4 to 6 days’, ‘7 to 10 days’, ‘11 to 20 days’, ‘more than 20 days’	Responses were grouped as ‘0 days’, ‘1–3 days’, or ‘4 or more days’ as a proxy for chronic absenteeism standards of an average of 15 days or more absent in a school year [16].
Suspensions	“How many times have you ever had an in school or out of school suspension?” Response options: ‘never’, ‘1 time’, ‘2 times’, ‘3 times’, ‘4 times’, ‘5 or more times’	Suspension was endorsed if the respondent indicated ‘1 time’ or more on this item.
Education aspirations	Respondents were asked “How far in school do you think you will get?” Response options: ‘complete Grade 8′, ‘go to high school (Grade 9 to Grade 12) but not complete’, ‘graduate from high school (Grade 12)’, ‘get a diploma or certificate from a trade, technical or vocational school or a business college, community college, or other non-university certificate program’, ‘graduate from university with a bachelor’s degree’, ‘graduate from university with a graduate degree (Master’s or PhD)’, ‘I don’t know’	Responses were combined into three categories: ‘high school or less’, ‘any post-secondary’, or ‘I don’t know’.
**Provincial Education** **Assessment**	**Competency Descriptions**	
Grade 3 numeracy	Predicts an element in a repeating pattern; Understands that the equals symbol represents an equality of the terms found on either side of the symbol; Understand that a given whole number can be represented in a variety of ways; Uses various mental mathematical strategies to determine answers to addition and subtraction questions up to the number 18.	‘Yes’ if ‘meeting or approaching’ on all four competencies
Grade 3 reading	Reflects on and sets reading goals; Uses strategies during reading to make sense of texts; Demonstrates comprehension.	‘Yes’ if ‘meeting or approaching’ on all three competencies
Grade 7 mathematics	Orders fractions; Orders decimal numbers; Understands that a given number may be represented in a variety of ways; Uses number patterns to solve mathematical problems; Uses a variety of strategies to calculate and explain a mental mathematical problem.	‘Yes’ if ‘meeting or approaching’ on all five competencies
Grade 7 student engagement	Demonstrates an interest in his/her learning; Engages in self-assessment; Aware of learning goals as a unit of study and/or personal learning goals; Participates in lessons; Accepts responsibility for assignments.	‘Yes’ if ‘established or developing’ on all five competencies
Grade 8 reading/writing	Understands key ideas and messages in a variety of texts; Interprets a variety of texts; Responds critically to a variety of texts; Generates, selects and organizes ideas to support the reader’s understanding; Chooses language (word choices and sentence patterns) to make an impact on the reader; Uses conventions (spelling, grammar, and/or punctuation) and resources to edit and proofread to make meaning clear.	‘Yes’ if ‘meeting or approaching’ on all six competencies
