# Supplementary material for: Adverse Childhood Experiences and Education Outcomes among Adolescents: Linking Survey and Administrative Data
Source: Int J Environ Res Public Health. 2022 Sep 14;19(18):11564. doi: 10.3390/ijerph191811564 (PMC9517426; doi:10.3390/ijerph191811564)
Supplement: Supplementary file 1 [file ijerph-19-11564-s001.zip › ijerph-1830826-supplementary.pdf]

Supplementary Table S1: Group difference between adolescents linked to administrative data at MCHP and those that did not consent to linkage

	**Linked (n= 840) %**	**Not linked (n= 162) %**	X^2 (df) / *t* (df)
**ACE history**			
No ACE	14.17	12.23	0.37 (1)
At least 1 ACE	85.83	87.77	
**Sex**			
Male	48.39	47.53	0.04 (1)
Female	51.61	52.47	
**Household income**			
$49,999 or less	20.30	24.31	5.68 (3)
$50,000-$99,999	35.89	42.36	
$100,00-$149,999	24.01	19.44	
$150,000 or more	19.80	13.89	
**Age**			
Years, mean (sd)	15.26 (1.07)	15.46 (1.07)	2.21 (999) *

*ACE = adverse childhood experience; *$p \leq .05$*

Supplementary Table S2: Odds and relative risk ratios of the independent variable and covariates in the adjusted regression models (Model 2 in Table 3) for the self-reported educational outcomes for adolescents with at least 1 ACE compared to those with no ACEs.

	Self-reported educational outcomes OR/RRR (95% CI)							
	**Grade repetition**	**School suspensions**	**Grades**		**Absenteeism**		**Educational Aspirations**	
**Model 2-adjusted**	**No (ref)/Yes**	**Never (ref)/ '1 or more'**	**'A' vs. 'C or lower'**	**'A' vs. 'B'**	**0 vs. '1-3 days'**	**0 vs. '4 or more days'**	**'Any post-secondary' vs. 'high school or less'**	**'Any post-secondary vs. 'I don't know'**
**ACE, ≥ 1**	1.72 (0.50, 5.93)	3.33*** (1.61, 6.92)	3.21** (1.42, 7.29)	1.11 (0.67, 1.84)	1.44 (0.91, 2.28)	2.45** (1.28, 4.68)	1.93 (0.92, 4.03)	3.34* (1.00, 11.13)
**Sex, female**	0.67 (0.37, 1.21)	0.23 *** (0.15, 0.34)	0.49 ** (0.34, 0.72)	0.62 ** (0.44, 0.88)	1.23 (0.88, 1.72)	1.40 (0.95, 2.08)	0.69 (0.47, 1.01)	1.38 (0.82, 2.3)
**Age**	1.90 *** (1.43, 2.53)	0.94 (0.79, 1.11)	1.09 (0.91, 1.30)	0.98 (0.83, 1.15)	1.03 (0.88, 1.21)	1.20 * (1.00, 1.43)	0.85 (0.71, 1.02)	0.64 *** (0.49, 0.82)
**Household income, < $50,000 (ref)**								
**$50,000-$99,999**	0.38 *** (0.19, 0.76)	.83 (0.52, 1.31)	0.50 ** (0.31, 0.79)	0.50 ** (0.31, 0.81)	1.24 (0.77, 2.00)	0.71 (0.43, 1.20)	0.45 *** (0.29, 0.71)	0.86 (0.47, 1.6)
**$100,00-$149,999**	0.15 ** (0.05, 0.47)	0.73 (0.43, 1.24)	0.29 *** (0.16, 0.51)	0.29 *** (0.16, 0.51)	1.04 (0.62, 1.73)	0.48 * (0.26, 0.86)	0.31 *** (0.18, 0.54)	0.51 (0.24, 1.1)
**$150,000 or more**	0.36* (0.14, 0.89)	0.45 ** (0.24, 0.85)	0.12 *** (0.06, 0.26)	0.34 *** (0.19, 0.60)	1.33 (0.76, 2.32)	0.81 (0.44, 1.51)	0.13 *** (0.06, 0.27)	0.20 ** (.07, .56)

ACE=Adverse Childhood Experience; OR= odds ratio; RRR=relative risk ratio, CI=confidence interval; [a] Stata computes relative risk ratio and not odds ratios for multinomial logistic regression*; *p≤ .05; **p≤ .01; ***p ≤ .001*

Supplementary Table S3: Odds ratios of the independent variable and covariates included in the adjusted regression analyses (Model 2 in Table 4) for the provincial educational assessment outcomes for adolescents with at least 1 ACE compared to those with no ACEs.

	**Provincial educational assessment outcomes OR (95% CI)**				
	**Grade 3 Numeracy Assessment (meeting/approaching)**	**Grade 3 Reading Assessment (meeting/ approaching)**	**Grade 7 Mathematics Assessment (meeting/ approaching)**	**Grade 7 Student Engagement Assessment (established/ developing)**	**Grade 8 Reading/ Writing Assessment (meeting/ approaching)**
**Model 2-adjusted**					
**ACE, ≥ 1**	0.83 (0.39, 1.73)	0.99 (0.41, 2.36)	0.50 (0.23, 1.09)	0.58 (0.30, 1.09)	1.04 (0.40, 2.69)
**Sex, male**	0.98 (0.61, 1.58)	0.94 (0.53, 1.65)	0.79 (0.53, 1.19)	0.45 (0.31, 0.67)	0.32 (0.17, 0.60)
**Household Income, < $50,000 (ref.)**					
**$50,000-$99,999**	2.57* (1.35, 4.86)	1.94 (0.93, 4.05)	1.45 (0.87, 2.43)	2.18 *(1.31, 3.64)	1.79 (0.91, 3.52)
**$100,00-$149,999**	2.37* (1.19, 4.70)	2.13 (0.95, 4.81)	2.90* (1.51, 5.55)	2.37* (1.33, 4.21)	6.12* (2.15, 17.41)
**$150,000 or more**	4.15* (1.79, 9.58)	5.72* (1.80, 18.16)	3.08 *(1.52, 6.24)	1.87* (1.03, 3.38)	5.89* (1.87, 18.53)
**Missing**	0.77 (0.28, 2.15)	1.34 (0.34, 5.18)	1.24 (0.46, 3.35)	1.32 (0.51, 3.43)	1.39 (0.37, 5.28)

*ACE=Adverse Childhood Experience; OR= odds ratio; CI=confidence interval; *$p \leq .05$*

Supplementary Tables: S4 & S5

Table S4: Odds or relative risk of educational outcomes for adolescents with 1, 2, 3 or 4+ ACEs compared to adolescents with no ACEs.

Educational outcome	Model 1 - unadjusted[a]				Model 2 - adjusted[a]			
	1 ACE	2 ACEs	3 ACEs	4+ ACEs	1 ACE	2 ACEs	3 ACEs	4+ ACEs
	OR (95% CI)				Adjusted OR (95% CI)			
Grade repetition								
No (reference)	1.00	1.00	1.00	1.00	1.00	1.00	1.00	1.00
Yes	1.04 (0.24-4.45)	1.04 (0.23-4.72)	2.38 (0.58-9.76)	5.21 (1.54-17.58)***	0.87 (0.20-3.84)	0.74 (0.15-3.54)	1.67 (0.38-7.36)	3.56 (0.94-13.50)
In-school or out-of-school suspension								
Never (reference)	1.00	1.00	1.00	1.00	1.00	1.00	1.00	1.00
1 or more	3.04 (1.41-6.56)**	2.30 (1.03-5.16)*	1.91 (0.79-4.60)	5.29 (2.53-11.06)***	3.01 (1.36-6.63)**	2.49 (1.07-5.80)*	2.01 (0.80-5.06)	6.82 (3.05-15.24)***
	RRR[b] (95% CI)				Adjusted RRR[b] (95% CI)			
Self-reported grades								
A (reference)	1.00	1.00	1.00	1.00	1.00	1.00	1.00	1.00
B	1.04 (0.59-1.85)	1.35 (0.75-2.42)	1.50 (0.79-2.85)	2.14 (1.23-3.73)**	0.83 (0.46-1.51)	1.10 (0.59-2.05)	1.22 (0.62-2.41)	1.86 (1.01-3.42)*
C or lower	2.81 (1.18-6.72)*	2.54 (1.02-6.34)*	4.29 (1.71-10.76)**	9.57 (4.18-21.93)***	2.20 (0.90-5.36)	1.78 (0.68-4.63)	3.25 (1.24-8.48)*	7.03 (2.91-17.01)***
Absenteeism (monthly)								
0 (reference)	1.00	1.00	1.00	1.00	1.00	1.00	1.00	1.00
1 to 3 days	1.27 (0.77-2.09)	1.01 (0.60-1.71)	1.55 (0.83-2.87)	2.74 (1.56-4.79)***	1.37 (0.81-2.32)	1.11 (0.64-1.94)	1.59 (0.83-3.05)	3.15 (1.68-5.90)***
4 or more days	1.74	1.53	4.09	8.57	1.70	1.18	3.51	7.83

	(0.85-3.59)	(0.72-3.24)	(1.86-8.98)***	(4.17-17.61)***	(0.81-3.57)	(0.54-2.60)	(1.55-7.96)**	(3.57-17.17)***
Educational aspirations								
High school or less	1.10 (0.47-2.60)	1.69 (0.73-3.92)	3.48 (1.52-7.96)**	6.03 (2.88-12.64)***	0.78 (0.32-1.91)	1.20 (0.49-2.93)	2.71 (1.13-6.49)*	4.45 (2.00-9.90)***
Any post-secondary (reference)	1.00	1.00	1.00	1.00	1.00	1.00	1.00	1.00
I don't know	2.86 (0.80-10.28)	2.26 (0.59-8.72)	3.32 (0.83-13.24)	6.21 (1.82-21.23)**	2.07 (0.56-7.63)	1.65 (0.41-6.62)	2.30 (0.55-9.53)	3.50 (0.96-12.79)

ACE= Adverse Childhood Experience; OR= odds ratio; CI=confidence interval; RRR=relative risk ratio; models adjusted for sex, age, and household income, [a] Reference group: No ACEs; [b] Stata computes relative risk ratio and not odds ratios for multinomial logistic regression; *p≤ .05; **p≤ .01; ***p ≤ .001

Table S5: Odds of educational outcomes for provincial assessments for adolescents with 1,2,3 and 4+ ACEs compared to adolescents with no ACEs.

Provincial Grade Level Assessments	No ACEs (reference)	1 ACE	2 ACEs	3 ACEs	4+ ACEs
Grade 3 Numeracy (Meeting/approaching)					
OR (95% CI)	1.00	1.09 (0.46, 2.61)	0.65 (0.28, 1.53)	0.61 (0.24, 1.57)	0.57 (0.26, 1.26)
AOR (95% CI)	1.00	1.16 (0.48, 2.84)	0.70 (0.29, 1.72)	0.68 (0.26, 1.83)	0.76 (0.32, 1.79)
Grade 3 Reading (Meeting/approaching)					
OR (95% CI)	1.00	1.10 (0.39, 3.07)	0.63 (0.23, 1.70)	0.90 (0.27, 3.00)	0.90 (0.34, 2.37)
AOR (95% CI)	1.00	1.26 (0.44, 3.58)	0.74 (0.26, 2.07)	1.13 (0.33, 3.90)	1.32 (0.46, 3.75)
Grade 7 Mathematics (Meeting/approaching)					
OR (95% CI)	1.00	0.62 (0.26, 1.49)	0.71 (0.28, 1.79)	0.40 (0.16, 1.01)*	0.29 (0.13, 0.65)*
AOR (95% CI)	1.00	0.70 (0.29, 1.68)	0.82 (0.32, 2.11)	0.48 (0.19, 1.24)	0.37 (0.16, 0.87)*
Grade 7 Student Engagement (Established/developing)					
OR (95% CI)	1.00	0.71 (0.35, 1.45)	0.70 (0.33, 1.48)	0.47 (0.22, 1.02)*	0.53 (0.27, 1.05)
AOR (95% CI)	1.00	0.75 (0.36, 1.55)	0.69 (0.32, 1.50)	0.48 (0.21, 1.09)	0.53 (0.25, 1.10)
Grade 8 Reading/Writing (Meeting/approaching)					
OR (95% CI)	1.00	1.04 (0.36, 3.04)	0.72 (0.25, 2.06)	0.47 (0.16, 1.39)	0.86 (0.31, 2.34)
AOR (95% CI)	1.00	1.33 (0.44, 4.04)	0.88 (0.29, 2.68)	0.69 (0.22, 2.22)	1.34 (0.45, 4.01)

ACE=Adverse Childhood Experience; OR= odds ratio; CI=confidence interval; adjusted for sex and household income;*p≤ .05
